# Genome-wide screening of novel RT-qPCR reference genes for study of GLRaV-3 infection in wine grapes and refinement of an RNA isolation protocol for grape berries

**DOI:** 10.1186/s13007-021-00808-4

**Published:** 2021-10-28

**Authors:** Yashu Song, Robert H. Hanner, Baozhong Meng

**Affiliations:** 1 Department of Molecular and Cellular Biology, University of Guelph, 50 Stone Road, Guelph, ON N1G2W1 Canada; 2 Department of Integrative Biology, University of Guelph, 50 Stone Road, Guelph, ON N1G2W1 Canada

**Keywords:** *Vitis vinifera*, Wine grapes, Viruses, Grapevine leafroll-associated virus 3, RNA-Seq, RT-qPCR, Gene expression and normalization, geNorm, NormFinder, BestKeeper, Virus–host interactions

## Abstract

**Background:**

Grapevine, as an essential fruit crop with high economic values, has been the focus of molecular studies in diverse areas. Two challenges exist in the grapevine research field: (i) the lack of a rapid, user-friendly and effective RNA isolation protocol for mature dark-skinned berries and, (ii) the lack of validated reference genes that are stable for quantification of gene expression across desired experimental conditions. Successful isolation of RNA with sufficient yield and quality is essential for downstream analyses involving nucleic acids. However, ripe berries of dark-skinned grape cultivars are notoriously challenging in RNA isolation due to high contents of polyphenolics, polysaccharides, RNase and water.

**Results:**

We have optimized an RNA isolation protocol through modulating two factors at the lysis step that could impact results of RNA isolation - 2-ME concentration and berry mass. By finding the optimal combination among the two factors, our refined protocol was highly effective in isolating total RNA with high yield and quality from *whole* mature berries of an array of dark-skinned wine grape cultivars. Our protocol takes a much shorter time to complete, is highly effective, and eliminates the requirement for hazardous organic solvents. We have also shown that the resulting RNA preps were suitable for multiple downstream analyses, including the detection of viruses and amplification of grapevine genes using reverse transcription-polymerase chain reaction (RT-PCR), gene expression analysis via quantitative reverse transcription PCR (RT-qPCR), and RNA Sequencing (RNA-Seq). By using RNA-Seq data derived from Cabernet Franc, we have identified seven *novel* reference gene candidates (CYSP, NDUFS8, YLS8, EIF5A2, Gluc, GDT1, and EF-Hand) with stable expression across two tissue types, three developmental stages and status of infection with grapevine leafroll-associated virus 3 (GLRaV-3). We evaluated the stability of these candidate genes together with two *conventional* reference genes (actin and NAD5) using geNorm, NormFinder and BestKeeper. We found that the *novel* reference gene candidates outperformed both actin and NAD5. The three most stable reference genes were CYSP, NDUFS8 and YSL8, whereas actin and NAD5 were among the least stable. We further tested if there would be a difference in RT-qPCR quantification results when the most stable (CYSP) and the least stable (actin and NAD5) genes were used for normalization. We concluded that both actin and NAD5 led to erroneous RT-qPCR results in determining the *statistical significance* and *fold-change values* of gene expressional change.

**Conclusions:**

We have formulated a rapid, safe and highly effective protocol for isolating RNA from recalcitrant berry tissue of wine grapes. The resulting RNA is of high quality and suitable for RT-qPCR and RNA-Seq. We have identified and validated a set of *novel* reference genes based on RNA-Seq dataset. We have shown that these new reference genes are superior over actin and NAD5, two of the *conventional* reference genes commonly used in early studies.

**Supplementary Information:**

The online version contains supplementary material available at 10.1186/s13007-021-00808-4.

## Background

Grapevine (*Vitis* spp.) is a major fruit crop with global cultivation of over 7.4 million hectares. It has high economic value in producing a variety of products, including table fruit, juice, seed oil and wine [1]. Over the past few decades, this woody fruit crop has been the focus of diverse molecular studies, including responses to abiotic or biotic stresses, developmental changes and impact on berry quality, and host–pathogen interactions. Studies often involved using nucleic-acid based assays such as RT-PCR and RT-qPCR [2–11], or high-throughput sequencing (HTS) such as microarray and RNA-Sequencing (RNA-Seq) [12–14]. While these are powerful technologies that deliver insights to our understanding of grapevine molecular biology, success of the analyses is heavily dependent on the integrity and yield of total RNA isolated from grapevine tissues. RNA isolation has been one of the major technical challenges for studies of woody plants, especially grapevine, due to the abundant presence of secondary metabolites such as polyphenols and polysaccharides [15]. This issue is especially acute for berries. Berries of dark-skinned grape cultivars, especially at veraison (i.e., the stage where berries start to change colour and soften) through harvest, are particularly problematic when it comes to the isolation of high quality RNA. Compared to other organs of grapevine, veraison and ripe dark-skinned berries have a low concentration of nucleic acids, but high levels of water content, soluble solids (e.g., glucose and fructose), lipids, and RNases, on top of the already abundant polyphenolic compounds and polysaccharides present in the tissues [15, 16]. High levels of these compounds interfere with RNA isolation, leading to the extracted RNA being low in concentration and poor in integrity [16, 17], making downstream analyses involving berries essentially impossible.

Extensive efforts have been made in the past to resolve this issue by developing RNA isolation protocols suitable for dark-skinned grapevine berries. However, these methods mostly involved the use of hazardous organic solvents such as phenol and chloroform [15, 17–22]. In addition, some of the methods are tedious and time-consuming as the protocols included many steps and long extraction time spanning two days or more. These protocols require repetitive centrifugations at controlled temperatures, overnight precipitation, and as a result, only a limited number of samples can be processed at a time [15, 17–22]. Some methods involved the extraction of total RNA from seeds, skin, or flesh of berries separately [15, 21, 23], which may not be applicable for downstream analyses that target *whole* berries, such as transcriptomic analysis of the whole berry under various biotic and abiotic conditions. For dark-skinned wine grapes with high values for wine-making and for the purpose of molecular studies, the lack of a robust RNA isolation protocol would hinder future molecular analyses concerning any berry-related questions. Development of a rapid, user-friendly and highly effective protocol that can be used for large-scale isolation of quality RNA from whole mature berries of dark-skinned grapevine cultivars is much needed.

Besides the challenges in RNA isolation, another problem constantly faced by grapevine researchers has been the lack of properly validated reference genes in RT-qPCR studies. Gene expression analysis via RT-qPCR is an essential approach taken by researchers to increase understanding of the dynamic molecular interplay in grapevine under various adverse conditions. Compared to conventional RT-PCR, RT-qPCR provides a more sensitive and accurate measurement of target transcripts [24]. As such, RT-qPCR has been used extensively in various gene expression studies [4, 25–29]. However, reliable quantification of gene expression using RT-qPCR is dependent on various factors, including the integrity and yield of RNA, efficiency of cDNA synthesis and PCR amplification, primer efficiency, difference in the initial sample amount, and variation in transcriptional activity in the investigated cells and tissues [30–32]. Reference genes, whose expression levels remain constant, at least ideally, regardless of experimental treatment, developmental stages, or type of tissues or cells, were therefore used as an internal control to normalize the variations of gene expression data brought by these variables in RT-qPCR analysis [30, 33–36].

Selection of proper reference genes that exhibit minimal changes in expression during a specific experiment is crucial for the accuracy of RT-qPCR analysis. However, past studies often selected reference genes based on assumptions rather than evidence [35, 37]. Genes involved in basic cellular processes, such as cell structure and primary metabolism, referred to as ‘housekeeping genes’, were presumed to have stable expression across tissues, cell types, and experimental conditions. These genes were of primary choice as RT-qPCR reference genes [15, 38]. Some of the most commonly used reference genes as reported in the literature include 18S rRNA, glyceraldehyde-3-phosphate dehydrogenase (GAPDH), translation elongation factor 1α (EF-1α), ubiquitin (UBQ), actin (ACT), alpha-tubulin (α-TUB), and β-TUB [38–41]. However, these ‘presumed’ *conventional* reference genes have been discredited by an increasing number of studies. A high degree of expressional variation among these genes was reported in diverse plant species, including *Arabidopsis* [38], rice [42], maize [43], tomato [44, 45], wheat [46], and poplar [47]. In addition, it has become clear that there are no universal reference genes that would work for all plant species, all tissue types, and different experimental conditions [30, 35, 48]. Therefore, it is necessary to first identify and validate reference genes with low expressional variation for each species of interest and for the intended research, before carrying out gene expression analysis with RT-qPCR [35, 49, 50].

A similar situation exists for gene expression and quantification studies involving grapevine. A majority of RT-qPCR studies pertaining to grapevine adopted a literature-based approach when searching for reference gene candidates [15, 37, 51–55]. Consequently, the identified candidates circled back to the ‘presumed’ *conventional* reference genes mentioned above; yet, their expressional stability remains questionable. The advent of RNA-Seq technologies has enabled genome-wide identification of reference genes with little or no expressional fluctuation in a designed experimental system. These *novel* reference genes were more credible than their *conventional* counterparts, as their identified stability was based on transcriptomic data validation, rather than presumption. Studies have used transcriptomic data derived from RNA-Seq or microarray to identify *novel* reference genes stably expressed in a range of plant species, including *Arabidopsis* [38], barley [56], wheat [46, 57, 58], soybean [39, 59], maize [60], tomato [44, 45], upland cotton [61] and poplar [47]. All of these *novel* reference genes were proven to have higher stability than the *conventional* reference genes described above [38, 45–47, 57–60, 62, 63]. For example, using transcriptomic data, Czechowski et al. [38] identified five novel reference genes: TIP41-like, two subunits of PP2A (At1g13320 and At1g59830), At4g33380, and a SAND family gene. Importantly, these novel reference genes outperformed *conventional* reference genes including ACT2, TUB6, EF-1α, UBQ10, and GAPDH by demonstrating less expressional fluctuation under various conditions tested (e.g., genotype, developmental stages, organ types, biotic, and abiotic stresses). Furthermore, these *novel* reference genes had higher stability rankings when examined by the bioinformatics tool, geNorm [38]. Two independent studies have used microarray data to search for the most stable *novel* reference genes of wine grape cultivars under biotic stress (i.e., infection with *Plasmopara viticola* and *Botrytis cinerea*) [64], or abiotic stress (water and heat stress) [65]. Two other studies have used RNA-Seq data to identify *novel* reference genes in berries of table grapes across different phenological stages and under abiotic stress [66, 67]. However, no studies have used genome-wide screening to identify reference genes with stable expression across viral infection, tissue types and phenological stages.

A peculiarity of grapevine is that it is susceptible to a wide spectrum of viral pathogens. More than 80 viruses and virus-like agents have been documented to infect grapevine [68], constituting the most substantial group of viruses that infect a single plant crop. Grapevine leafroll-associated virus 3 (GLRaV-3) is a positive-sense, single-stranded RNA (ssRNA) virus of the *Ampelovirus* genus within the family *Closteroviridae* [69]. It is one of the major grapevine viruses with a worldwide prevalence and is the main agent associated with grapevine leafroll disease (GLRD); the most prevalent and destructive viral disease of grapevine that afflicts global grape and wine production [70–72]. Infection with GLRaV-3 can result in significant yield reduction and altered fruit chemistry that negatively impact the quality of fresh fruits, juice and wine, as well as the profitability and lifespan of vineyards [71, 73]. There are limited studies on the impact of GLRaV-3 infection on the global gene expression of grapevine, which limits our understanding of the pathogenesis and pathology of GLRaV-3 as well as the complex biology of GLRD. Preliminary results revealed that GLRaV-3 infection induced up-regulation of sugar transporter genes in leaves but down-regulation in berries at veraison and harvest [5, 13]. In addition, genes of flavonoid biosynthetic pathway were differentially regulated in leaves and berries of dark-skinned cultivars infected with GLRaV-3, leading to de novo synthesis of anthocyanins in GLRaV-3-infected leaves - hence, the reddish-to-purple discolouration of leaf blades [4]. In contrast, GLRaV-3 infection led to reduced levels of flavonoids in berries including anthocyanins, proanthocyanidins, and flavonols [5, 74–76]. Nevertheless, we are still at the exploration phase of understanding molecular mechanisms underlying the pathological impacts of GLRaV-3 on grapevine. Identification of reference genes whose expression remains stable in GLRaV-3-infected grapevine will open doors for future research on GLRaV-3-host interactions at transcriptomic level.

In this study, we aimed to address two key issues related to current research on gene expression and virus–host interactions in grapevine. First, we have developed an optimized RNA isolation protocol suitable for mature berries of dark-skinned wine grape cultivars. This protocol produced a much higher yield of RNA with high integrity, making it suitable for downstream analyses such as RNA-Seq and RT-qPCR. This is the first total RNA isolation protocol that can be used on whole ripe red berries without the need for hazardous organic solvents and can be completed within a much shorter time frame than the other protocols currently available. Second, using RNA-Seq data derived from two tissue types and three developmental stages of Cabernet Franc and a holistic bioinformatics approach, we have identified a set of *novel* reference genes whose expression is stable in different tissues, developmental stages and under viral infections.

## Material and methods

### Sample collection

Leaves and berries of *Vitis vinifera* cv. Cabernet Franc (clone 210 grafted on rootstock 3309) were collected in 2019 from a commercial vineyard located in Niagara Peninsula, Ontario, Canada. Prior to sample collection, viral infection status of vines was tested by RT-PCR using primers specific for a set of grapevine viruses that are included in the international certification programs, including five viruses associated with grapevine leafroll (GLRaV-1, -2, -3, -4, -7), three involved in rugose wood disease complex (GRSPaV, GVA, and GVB), four involved in the infectious degeneration and decline (GFLV, ArMV, TRSV, and TomRSV), plus GRBaV and GPGV [9].

For sample collection, fully expanded mature leaves were collected. The maturity of leaf samples was judged by chlorophyll content via SPAD meter [77]. SPAD meter emits both red light with 650 nm wavelength and infrared light of 940 nm [78]. Leaf chlorophyll absorbs red light but not infrared light. Therefore, by calculating the difference between transmittance values of 950 nm and 650 nm, the SPAD meter generates a chlorophyll content index (CCI) that is proportional to the chlorophyll content of the leaf [78]. For each vine (biological replicate), two fully expanded leaves with the highest CCI compared to the rest of the leaves of the same shoot were collected; berries of the same cluster were collected. Leaf and berry samples were collected from three vines tested positive for GLRaV-3 (three GLRaV-3-infected biological replicates) and three vines tested negative for the virus (three control biological replicates). Leaf samples were collected from each of the six vines at two developmental stages, E-L 31 (marked by pea-sized berries) and E-L 35 (veraison), based on the modified Eichhorn and Lorenz (E-L) system proposed by Coombe [79, 80]. Berry samples were collected at three developmental stages, E-L 31, E-L 35, and E-L 38 (harvest). Ripe berries at E-L 38 were collected from additional nine dark-skinned wine grape cultivars, including *V. vinifera* (Pinot Noir, Pinot Meunier, Gamay, Cabernet Sauvignon, and Merlot) and French-American hybrid grapes (De Chaunac, Marechal Foch, Chambourcin, and Baco Noir). All samples were immediately frozen in liquid nitrogen, then stored in dry ice for transportation. All samples were ground into fine powdery in liquid nitrogen using a mortar and a pestle, then stored under − 80 °C until further analysis.

### Total RNA isolation

RNA isolation protocol was optimized by adjusting two parameters at the lysis step: concentration of β-mercaptoethanol (2-ME) and sample mass. For leaf samples of both developmental stages and young berries at E-L 31, 50 mg of leaf sample and 100 mg of berry sample were used for total RNA isolation using the method developed recently in our lab [81]. For berries collected at E-L 35 and E-L 38, total RNA was isolated using the Spectrum™ Plant Total RNA Kit (Sigma-Aldrich) with the following modifications. Different amounts of 2-ME were added to the lysis buffer to test the effects on both the yield and quality of the total RNA. Also, the ground berry tissue powder used for RNA isolation was increased in increments. Tissue powders in the modified lysis buffer were subjected to further and vigorous grinding using a mortar and a pestle. The rest of RNA isolation steps followed the instruction from the vendor, except for the last washing step, where RNA-binding columns were washed three times instead of two.

Total RNA concentration, OD260/280 ratio, and OD260/230 ratio were assessed by NanoDrop ND-1000 spectrophotometer (Thermo Fisher Scientific, Wilmington, DE, USA). RNA integrity of all samples was verified through 1% agarose gel electrophoresis followed by staining with ethidium bromide. RNA integrity of leaf and berry samples collected from Cabernet Franc was further validated using Agilent 2100 Bioanalyzer via Novogene (Sacramento, CA, USA).

### RT-PCR

To examine the robustness of our optimized RNA isolation protocol in downstream analyses, total RNAs of Cabernet Franc (both leaf and berry samples) were used in RT-PCR with GLRaV-3-specific primers designed in our lab [2]. Total RNAs isolated from ripe berries of additional nine dark-skinned wine grape varieties (the infection status for GLRaV-3 was unknown) were used in RT-PCR for the amplification of phytoene desaturase (PDS) gene using primers PDS-853F and PDS-1252R, with an expected amplicon size of 500 bp. cDNA of all samples was synthesized with 5 μg of total RNA using Applied Biosystem High-Capacity cDNA Reverse Transcription Kit (Thermo Fisher Scientific, Carlsbad, CA, USA) according to manufacturer’s instructions.

### RNA-Seq analysis and identification of candidate reference genes

Total RNAs of leaf and berry samples from Cabernet Franc were sent to Novogene (Sacramento, CA, USA) for 150 bp paired-end mRNA sequencing on the NovaSeq 6000 platform. Raw RNA-Seq data was trimmed by Trim Galore! [82] to remove adaptor sequences and low-quality reads, followed by quality evaluation using FastQC [83]. Reads were mapped to the grapevine reference genome [*V. vinifera* cv. PN40024 [84] and transcript expressions were normalized to Transcripts Per Million (TPM) using Salmon [85].

Genes that were *non-differentially* expressed across the three developmental stages (E-L 31, 35, and 38), infectious status (tested positive or negative for GLRaV-3) and tissue types (leaf and berry) were identified as candidate reference genes using the TPM method described by Li et al. [86]. For detailed explanation of the TPM method, readers are referred to Li et al. [86]. Briefly, all four criteria below must be met for a gene to be considered a candidate: (1) relatively high levels of expression (mean [log_2_(TPM_gene_)] > 5); (2) low expressional variance across samples (standard-deviation [log_2_ (TPM_gene_)] < 1); (3) no sudden differential expression in any single sample (log_2_(TPM_gene_) – mean [log_2_(TPM_gene_)] < |2|); and (4) genes meeting all of the above criteria and shared by both leaf and berry RNA-Seq datasets were chosen as the *core-set of reference gene candidates* for our RT-qPCR analysis. The *core-set reference gene candidates* were further ranked by coefficient of variance (CV = standard deviation divided by mean) from the lowest to the highest. The top seven candidates with the lowest CV values were selected for further validation of their expressional stability by downstream statistical approaches.

### Function enrichment analysis

Functional enrichment analysis and pathway analysis were conducted to further understand the biological functions of the identified *core-set candidate reference genes*. Gene ontology (GO) and Kyoto Encyclopedia of Genes and Genomes (KEGG) enrichment analysis of the *core-set of candidate reference genes* were performed using DAVID with the default algorithm using an FDR cutoff at 0.01 [87]. GO consortium vocabularies were used for gene annotation and for grouping of the *core-set of candidate reference genes* at three levels, Biological Process (BP), Molecular Functions (MF), and Cellular Component (CC). KEGG annotations were used for pathway enrichment analysis.

### Validation of select candidate reference genes

RT-qPCR was performed on each of the seven selected *novel* reference gene candidates. Actin and NAD5, two of the *conventionally* used reference genes reported as the most suitable reference genes from an earlier study involving grapevines infected with GLRaV-3 [4], were included here in order to compare their expression stability with the seven *novel* reference gene candidates identified in this work (Table 1). Expression stability of these nine candidate reference genes was evaluated using three independent analytical methods: geNorm [33], NormFinder [30], and BestKeeper [88].

**Table 1:** Genes and primer sets used for RT-qPCR

Gene abbreviation	Accession no.	NCBI gene description	Primer sequence (5′–3′)	Amplicon size (bp)	Amplification efficiency (E)
EF-Hand	NM_001280964.1	EF-hand calcium-binding protein	F: TTTGACAGGGACCGTAGTGG R: GTCAGCCCCTTTACCGTGAG	188	98.25
GDT1	XM_019218145.1	GDT1-like protein 5	F: GGCTGCTCCAAACCTGTTGTC R: ACCTTGCTATCCCCTTTGGC	192	104.68
CYSP	NM_001281060.1	Cysteine protease	F: AAAATCAGGGTTCGTGTGGGTC R: GCAGTGTTCATCAGCCCACC	190	96.79
NDUFS8	XM_003631606.3	NADH dehydrogenase [ubiquinone] iron-sulfur protein 8 (mitochondrial)	F: CCGTAGAACGACCAGGTACGAC R: GCAATCTCGGTTTCCCAGCG	189	94.66
EIF5A2	XM_002285469.4	Eukaryotic translation initiation factor 5A-2	F: CCGCAAGAACGGCTACATCG R: CGGGTAACATGTGGAACATCAC	183	103.45
Gluc	XM_010651552.1	Endo-1,3;1,4-beta-d-glucanase	F: GCTTTTGCTGGGGTGCCAAG R: TGTTTCACGAGTGCCGGTG	175	95.74
YSL8	XM_002283586.3	Thioredoxin-like protein YLS8	F: TCAGGCGTGAAGAGAGAAAGC R: AGCCAGAACTTCATCCATCTGC	186	97.28
Actin	XM_002277287.4	Actin 1	F: ATCAGGAAGGACCTCTATGG R: ATCCACATCTGCTGGAAGG	206	92.98
NAD5	GU585873.1	NADH dehydrogenase subunit 5	F: GATGCTTCTTGGGGCTTCTTGTT R: CTCCAGTCACCAACATTGGCATAA	181	92.08
UFGT	XM_010659535.2	Anthocyanidin 3-*O*-glucosyltransferase 2	F: TCTTCCCTTCTGTGGTGCTTG R: TTATTGAGCAGGGGTCCAACAG	187	99.10

Seven *novel* reference genes candidates identified from our own RNA-Seq data were EF-hand, GDT1, CYSP, NDUFS8, EIF5A2, Gluc, YSL8. Two *conventional* reference genes used by a past study, Gutha et al. [4] were actin and NAD5. UFGT, shown previously as a responsive gene whose expression was influenced by GLRaV-3 infection, was chosen in this study for RT-qPCR validation

Target-specific primers were designed using Primer-BLAST [89] with a melting temperature (Tm) between 59 and 63 °C, primer length of 20–22 nucleotides, and amplicon size between 175 and 206 bp (Table 1). RT-qPCR was conducted in a 96-well plate using StepOnePlus Real-Time PCR System (Applied Biosystems, Foster City, CA, USA). Each 15 μL reaction contained 5 μL of fivefold diluted cDNA, 7.5 μL of Bio-Rad SoAdvanced™ Universal IT SYBR Green Supermix and 0.6 μL each of 10 μM primers. Reactions were run using cycling parameters of 98 °C for 3 min, 40 cycles of 98 °C for 10 s and 60 °C for 30 s, followed by a single cycle at 95 °C for 15 s, 75 °C for 1 min, and 95 °C for 15 s for melt curve analysis. RT-qPCR analysis for each sample was performed in triplicate. No-template controls were included for each RT-qPCR analysis. For each gene, the melting curve analysis was carried out to verify the specificity of amplification. Amplification efficiency (E) of each candidate reference gene/primer set was calculated based on a standard curve generated using fivefold dilution series (1; 1:5; 1:25; 1:125; 1:625). The standard curve was generated using StepOnePlus Software v2.3 (Applied Biosystems, Foster City, CA, USA). All primer pairs had amplification efficiency within the acceptable range, from 92.1 to 104.7% (Table 1). The same batch of diluted cDNA obtained from each biological replicate (control and GLRaV-3-infected leaf at E-L 31 and 35, control and GLRaV-3-infected berry at E-L 31, 35 and 38) was used as the template for RT-qPCR analysis to avoid potential impact of inconsistency in reverse transcription on downstream quantification of candidate reference genes.

## Results

### Leaf and berry sampling conditions

**Fig. 1:**
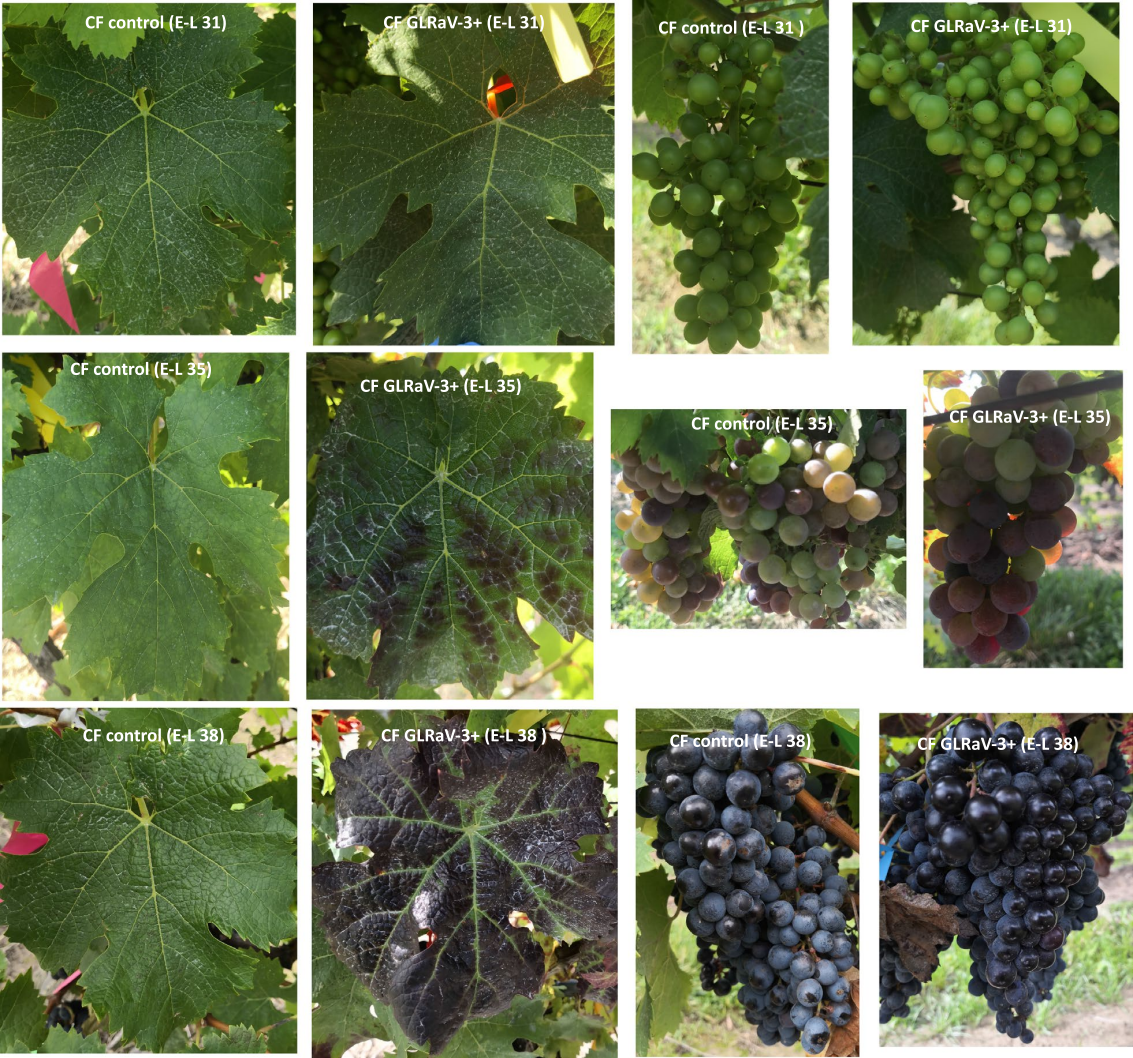
Condition of leaf and berry of control and GLRaV-3-infected vines at stages E-L 31, E-L 35, and E-L 38

Fully expanded mature leaves of vines (control and GLRaV-3-infected) at stage E-L 31 and E-L 35 and whole berries of vines (control and GLRaV-3-infected) at E-L 31, E-L 35, and E-L 38 were collected for this study. No observable difference was found between leaves of control vines and those of GLRaV-3-infected vines at E-L 31 (Fig. 1). At E-L 35 stage, we observed typical GLRD symptoms in GLRaV-3-infected leaves, characterized by red to purple pigmentation of interveinal regions (Fig. 1). GLRD symptoms progressed as vines grew into E-L 38 (harvest), with more leaves showing symptoms and more evident discoloration together with the development of downward curling on margins of mature leaves (Fig. 1). No physiological difference was observed between berries from control vines and those from GLRaV-3-infected vines at either E-L 31, E-L 35, or E-L 38. Berries collected at E-L 31 were green and hard, as expected. Berries collected at E-L 35 were softened to touch compared to E-L 31; in addition, a portion of berries in the same cluster started to change colour from green to purple (Fig. 1). At E-L 38, ripe berries showed dark-purple colour (Fig. 1) and emitted strong aroma.

### The refined RNA isolation method yielded quality RNAs suitable for downstream assays

Our lab had previously improved the Sigma kit and made it suitable for isolation of quality RNA from old grapevine leaves with GLRD symptoms by addition of 2.5% of polyvinylpyrrolidone (PVP-40) to the lysis buffer [81]. However, this modification failed to generate sufficient amounts of quality RNA from dark-skinned berry samples. We further identified two additional factors as potential variables that would impact the quality and yield of total RNA for dark-skinned berries: concentration of 2-ME and the amount of berry tissue used.

We first tested the ‘baseline protocol’ on ripe berries of Cabernet Franc using lysis buffer containing 1% of 2-ME and 50 mg of tissue, which was recommended by the vendor, and a modified lysis buffer (adding 2.5% PVP-40). Three biological replicates of ripe berries (i.e., berries collected from three vines) were used for each round of the test. NanoDrop results, including OD260/280 and OD260/230 ratios and RNA concentration (ng/μL), were recorded as the average of the three replicates. Very little RNA was obtained when the ‘baseline protocol’ was used (Additional file 1: Table S1, “Berry mass and 2-ME conc.” sub-sheet). We then preliminarily assessed the association between RNA yield/purity and the two variables identified in this study (berry mass and 2-ME concentration) using NanoDrop. We found that berry mass and 2-ME concentration were positively correlated with both the yield and purity of RNA from mature dark-skinned berry (Additional file 1: Table S1, “Berry mass and 2-ME conc.” sub-sheet). Based on the trend given by NanoDrop results, we have noted that increasing berry mass to 300–500 mg and 2-ME concentration to 2–3% at lysis step played a crucial role for optimal RNA isolation from dark-skinned berries (Additional file 1: Table S1, “Berry mass and 2-ME conc.” sub-sheet).”

We further tested the effectiveness of RNA isolation involving the identified parameters (300 mg berry mass, 2% of 2-ME, and 2.5% of PVP-40) on leaf and berry samples of Cabernet Franc at three different phenological stages (E-L 31, 35, and 38), as well as mature berries at E-L 38 of nine additional dark-skinned wine grape varieties, including five *V. vinifera* cultivars (Cabernet Sauvignon, Pinot Noir, Pinot Meunier, Gamay, and Merlot) and four hybrids (Marechal Foch, De Chaunac, Chambourcin, and Baco Noir). A consistent OD260/280 ratio of > 2.0 and OD260/230 ratio of > 1.9, and satisfying RNA yields ranging from 68.1 to 295.7 ng/μL were obtained for total RNA extracted from both berry samples collected at veraison and harvest (Additional file 1: Table S1, “Cab. Franc leaf and berry” and “Other nine grapevine varieties” sub-sheets).

**Fig. 2:**
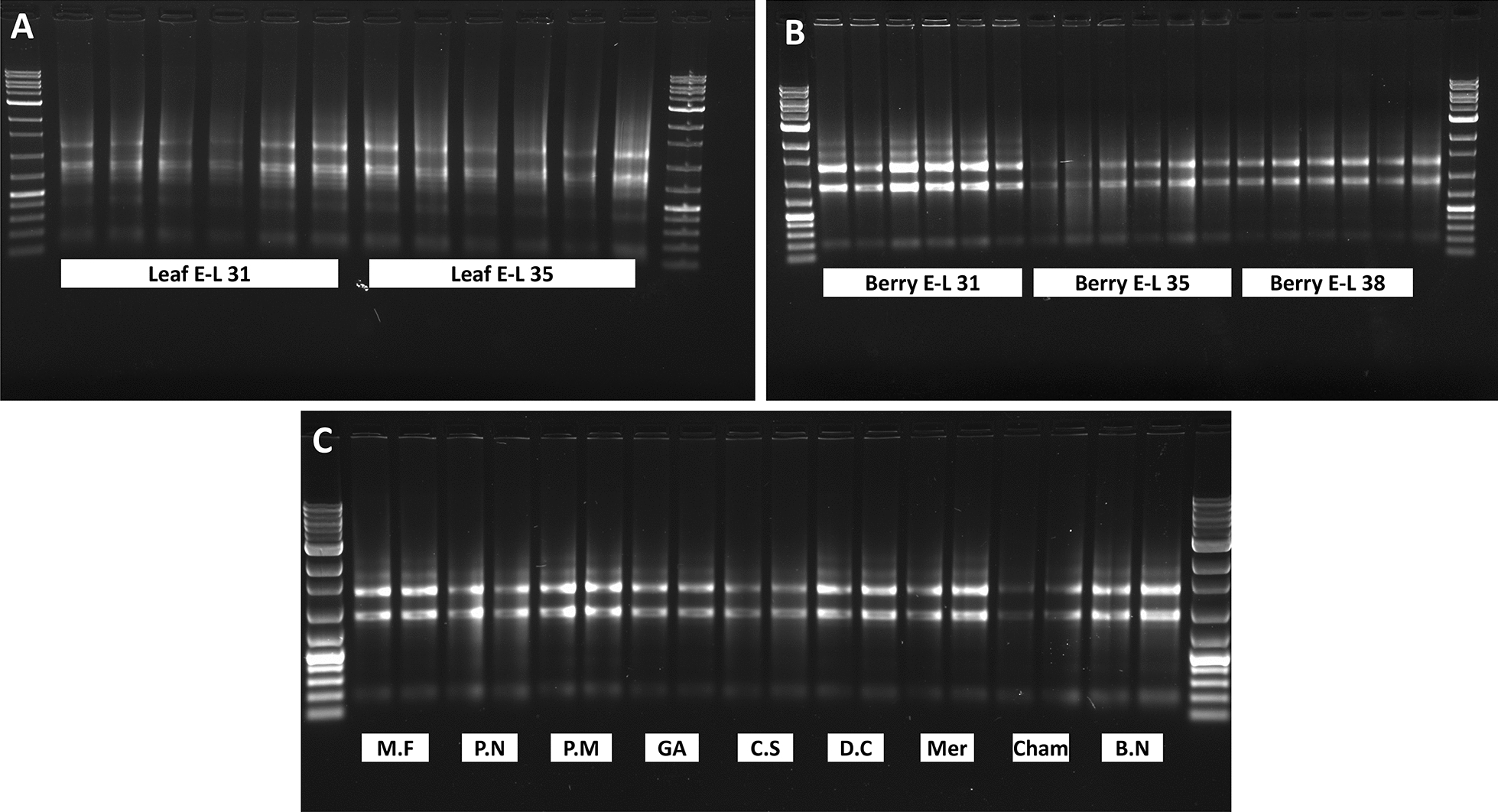
Profile of total RNA samples through electrophoresis on 1% agarose gels. **A** Total RNA extracted from leaf samples of Cabernet Franc collected at E-L 31 (six lanes on the left) and at E-L 35 (veraison) (six lanes to the right). **B** Total RNA extracted from berry samples of Cabernet Franc collected at E-L 31 (six lanes on the left), E-L 35 (six lanes in the middle), and E-L 38 (six lanes to the right). **C** Total RNA extracted from mature berries at E-L 38 of nine additional dark-skinned wine grape cultivars (two lanes per cultivar). M.F. = Marechal Foch; P.N. = Pinot Noir; P.M. = Pinot Meunier; GA = Gamay; C.S = Cabernet Sauvignon; D.C. = De Chaunac, Mer = Merlot; Cham = Chambourcin; and B.N. = Baco Noir

**Fig. 3:**
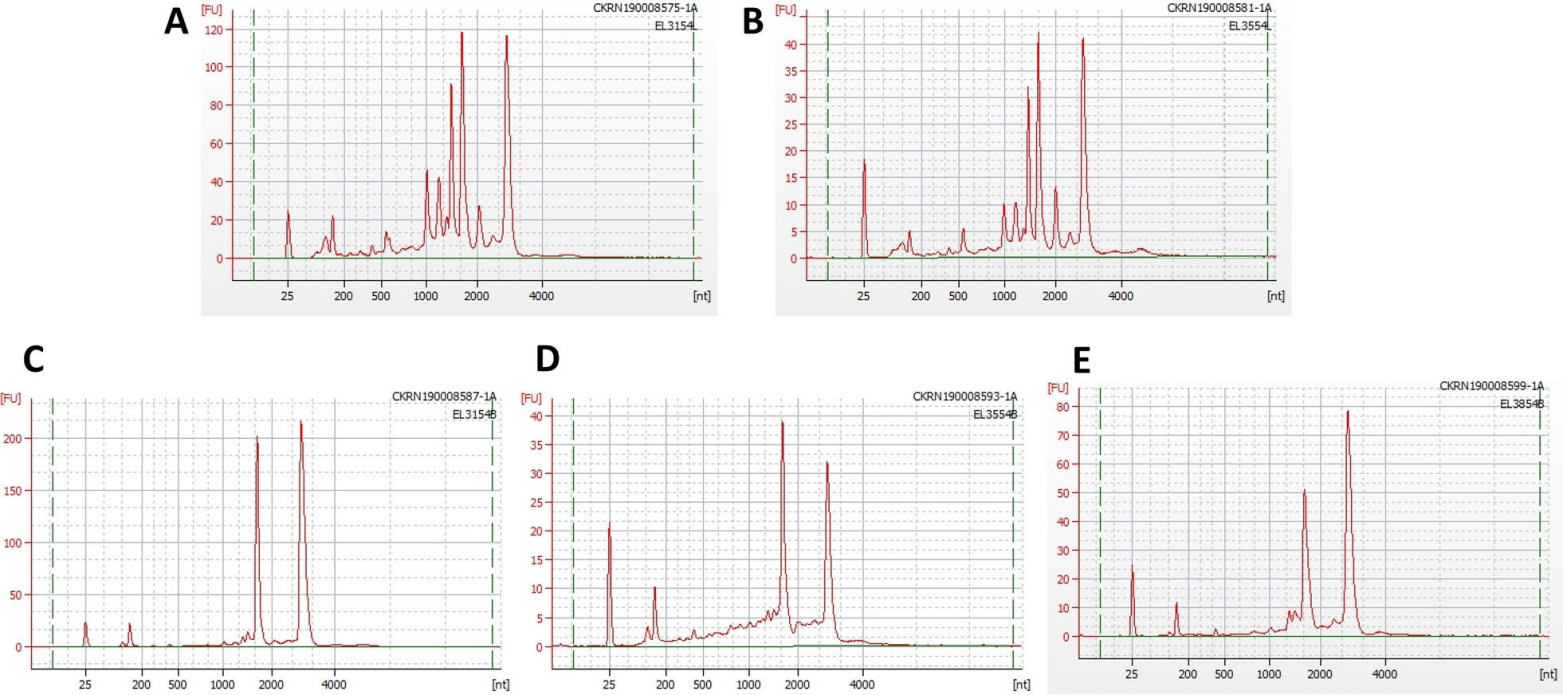
Quality of total RNA samples isolated from Cabernet Franc collected at three developmental stages as revealed by Agilent 2100 Bioanalyzer. **A** E-L 31 leaf; **B** E-L 35 leaf; **C** E-L 31 berry; **D** E-L 35 berry; **E** E-L 38 berry. All samples passed quality control and met the criteria required for RNA Sequencing (RNA-Seq)

The integrity of total RNA preps was examined through gel electrophoresis. Results showed that the RNA preps from both leaf and berry samples of Cabernet Franc collected at all three developmental stages (E-L 31, 35, and 38), and from ripe berries of additional nine dark-skinned wine grape varieties were intact (Fig. 2). The integrity and concentration of total RNAs isolated from leaf and berry samples of Cabernet Franc were further analyzed by using Agilent 2100 Bioanalyzer. All RNA samples passed quality control for the purpose of RNA-Seq with RNA integrity number (RIN) between 6.2 and 9.9 and concentrations between 76.15–517.93 ng/μL (Fig. 3 and Additional file 2: Table S2).

**Fig. 4:**
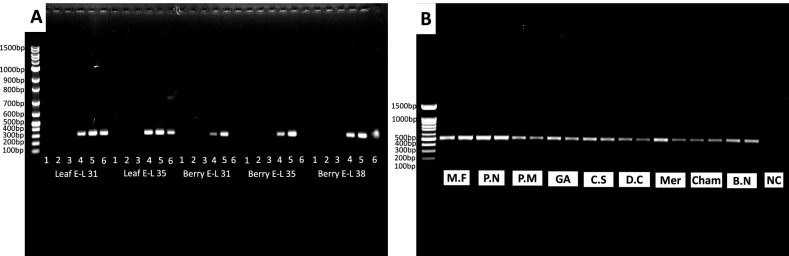
RT-PCR of Grapevine leafroll-associated virus 3 (GLRaV-3) and grapevine phytoene desaturase synthase (PDS) using total RNAs extracted from leaf and berry samples of dark-skinned wine grape cultivars. **A** Detection of GLRaV-3 in leaf and berry samples of Cabernet Franc. Lanes 1–3: samples free of GLRaV-3; lanes 4–6: samples infected with GLRaV-3; **B** detection of PDS from mature berries collected from nine dark-skinned wine grape varieties at E-L 38. Two samples for each cultivar were included in the test. Fainter bands for berry samples in lane 6 of each set in Fig. 4A were due to lower virus titer in this vine. M.F. = Marechal Foch; P.N. = Pinot Noir; P.M. = Pinot Meunier; GA = Gamay; C.S = Cabernet. Sauvignon; D.C. = De Chaunac, Mer = Merlot; Cham = Chambourcin; B.N. = Baco Noir; NC = negative control

Total RNA preparations of all samples were tested for their suitability for downstream analyses. RNAs isolated from leaf and berry samples of Cabernet Franc at different developmental stages were tested for the presence of GLRaV-3 via RT-PCR. As expected, samples collected from vines infected with GLRaV-3 all tested positive for the virus, regardless of developmental stages. In contrast, samples from GLRaV-3-free vines all tested negative as expected (Fig. 4A). Note that the lower intensity of PCR band in lane 6 of each set of berry samples of Fig. 4A was associated with a lower viral titer present in berries of this particular vine. Total RNAs isolated from ripe berries of the additional nine dark-skinned wine grape cultivars were used as templates for RT-PCR amplification of phytoene desaturase (PDS). DNA products of PDS with the expected size were successfully generated (Fig. 4B). PDS is often used as a positive control in RT-PCR for its ‘housekeeping’ expression. Successful amplification of PDS indicated the presence of mRNA corresponding to PDS; hence, the robustness of RNA isolation. Total RNAs isolated from leaf and berry samples of Cabernet Franc at different developmental stages were subjected to RNA-Seq at Novogene, with successful results (not published). Together, these results demonstrated that total RNAs obtained using our refined protocol were of high integrity and yield and are suitable for both RT-PCR and RNA-Seq analyses.

### Functional enrichment analysis

Using the TPM filtration method based on Li et al. [86], we have identified a total of 3402 and 3079 candidate reference genes in RNA-Seq datasets derived from berry and leaf samples, respectively (Additional file 3: Tables S3, S4). 2000 candidates were shared by both berry and leaf datasets and were therefore defined as the *2000 core-set reference gene candidates* (Additional file 3: Table S5). These reference gene candidates were preliminarily ranked for their expressional stability based on CV values, from the lowest (most stable) to the highest (least stable). Subsequently, GO enrichment and pathway analyses were conducted to understand their functional role in relation to host-virus interactions in different tissues at various developmental stages. GO analysis showed that a majority of these genes were over-represented in GO terms such as translation, nucleosome assembly, ATP hydrolysis/proton transport, tricarboxylic acid (TCA) cycle, protein folding, and ubiquitin-dependent protein catabolic process (Table 2). Results from KEGG essentially support those from GO analysis (Additional file 4: Table S6).

**Table 2:** Significantly enriched GO terms of the 2000 core reference genes candidates of *Vitis vinifera* cv. Cabernet Franc

ID code	Term	Gene number	P-value	FDR
GO biological process				
GO:0006412	Translation	95	1.26E−19	7.17E−17
GO:0002181	Cytoplasmic translation	23	1.12E−11	3.20E−09
GO:0006334	Nucleosome assembly	17	3.62E−07	6.87E−05
GO:0006446	Regulation of translational initiation	11	2.66E−06	3.25E−04
GO:0001731	Formation of translation preinitiation complex	12	2.86E−06	3.25E−04
GO:0015991	ATP hydrolysis coupled proton transport	14	1.25E−05	0.00118285
GO:0000027	Ribosomal large subunit assembly	13	4.62E−05	0.0037529
GO:0006099	Tricarboxylic acid cycle	12	6.41E−05	0.00456226
GO:0006457	Protein folding	29	1.04E−04	0.00655506
GO:0043161	Proteasome-mediated ubiquitin-dependent protein catabolic process	30	1.44E−04	0.00760634
GO:0006511	Ubiquitin-dependent protein catabolic process	18	1.47E−04	0.00760634
GO molecular function				
GO:0003735	Structural constituent of ribosome	118	5.84E−32	2.45E−29
GO:0003743	Translation initiation factor activity	28	7.11E−15	1.49E−12
GO:0000166	Nucleotide binding	52	1.84E−12	2.57E−10
GO:0004298	Threonine-type endopeptidase activity	14	1.13E−10	1.18E−08
GO:0003723	RNA binding	55	7.97E−08	6.68E−06
GO:0003676	Nucleic acid binding	47	1.65E−05	0.00115072
GO:0046961	Proton-transporting ATPase activity, rotational mechanism	11	4.34E−05	0.00259685
GO:0003746	Translation elongation factor activity	10	1.80E−04	0.00885122
GO:0031625	Ubiquitin protein ligase binding	16	1.90E−04	0.00885122
GO cellular component				
GO:0022625	Cytosolic large ribosomal subunit	61	1.44E−34	2.85E−32
GO:0022627	Cytosolic small ribosomal subunit	31	1.99E−15	1.96E−13
GO:0033290	Eukaryotic 48S preinitiation complex	13	6.26E−08	4.11E−06
GO:0016282	Eukaryotic 43S preinitiation complex	12	1.23E−07	6.08E−06
GO:0005747	Mitochondrial respiratory chain complex I	11	2.31E−07	7.58E−06
GO:0005852	Eukaryotic translation initiation factor 3 complex	11	2.31E−07	7.58E−06
GO:0000786	Nucleosome	19	3.37E−07	9.47E−06
GO:0005737	Cytoplasm	145	1.73E−06	4.26E−05
GO:0019773	Proteasome core complex, alpha-subunit complex	8	4.01E−06	8.77E−05
GO:0005829	Cytosol	74	8.02E−05	0.00157964
GO:0030687	Preribosome, large subunit precursor	12	5.62E−04	0.00959393
GO:0071004	U2-type prespliceosome	7	5.84E−04	0.00959393

*FDR* false discovery rate

Seven candidate reference genes with the lowest CV values were selected from the list of the *core-set reference gene candidates* for further validation of their expression stability (Table 1 and Additional file 3: Table S5). These seven *novel* reference gene candidates are YLS8, EIF5A2 CYSP, NDUFS8, Gluc, GDT1, and EF-hand (Table 1). The seven reference gene candidates were annotated using GO terms via PANTHER [90]. Briefly, YLS8, thioredoxin-like protein, was predicted to be located in the nucleus, likely serving as a component of the spliceosome [the U4/U6 xU5 tri-small nuclear ribonucleoproteins (snRNP) complex] that functions in pre-mRNA splicing. EIF5A2 is a member of the translation initiation factor 5A family and is involved in the initiation of translation. CYSP is a member of the C1 family of cysteine peptidases. It is associated with lysosome and is involved in protein degradation. NDUFS8, short for NADH dehydrogenase [ubiquinone] iron-sulfur protein 8 (mitochondrial), is a component of the complex I of the respiratory chain and is involved in electron transport. Gluc (endo-1,3;1,4-beta-d-glucanase) has hydrolase activities. It is involved in the breakdown of glucan polymers and thus plays a role in plant cell wall loosening. GDT1, GDT1-like protein 5, is located in the Golgi apparatus and is involved in ion transport across membrane. Little is known about the function of EF-hand, though it was reported to be involved in calcium ion binding.

### Expression stability of reference gene candidates

**Fig. 5:**
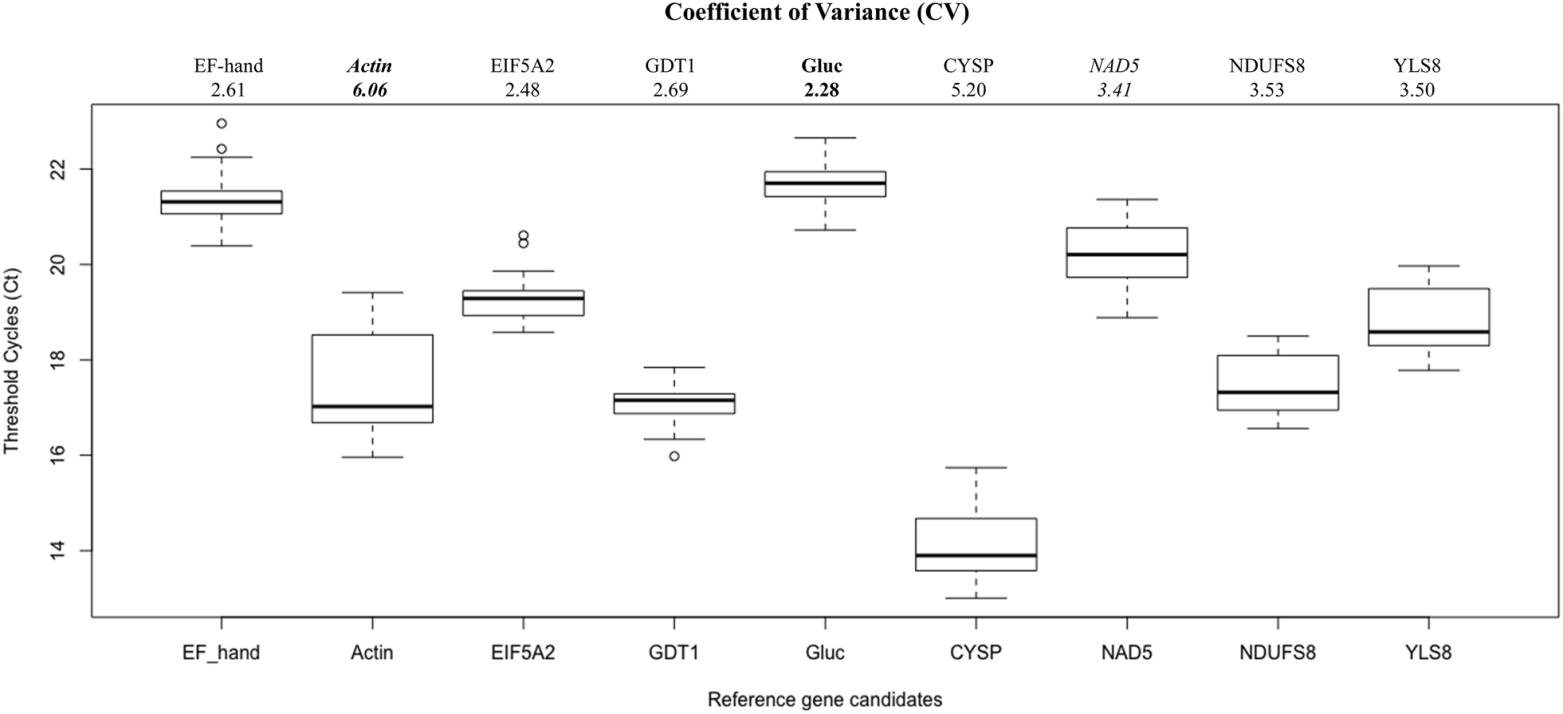
Expression profile of reference gene candidates of grapevine samples. For each reference gene candidate, absolute Ct values from all sample groups were combined. Sample groups comprised two tissue types (leaf and berry) at three developmental stages (E-L 31, E-L 35 and E-L 38), infected with and free from GLRaV-3. Each sample group has three biological replicates. The boxes indicate the range of scores between the 25th and the 75th percentiles. The thick line within each box represents the median. Maximum and minimum values are denoted by whiskers. Small circles represent outliers. Reference gene candidates with the highest CV (actin) and the lowest CV (Gluc) are highlighted in bold

Absolute Ct values of the seven *novel* reference gene candidates and two *conventional* reference genes (actin and NAD5) from all biological samples were collected and used to plot the expression profile chart (Fig. 5). These nine candidates had mean Ct values ranging from 14.08 (for CYSP) to 21.67 (for Gluc) (Fig. 5), suggesting that all candidates were expressed at moderate to high levels - a desired trait of genes as internal reference for RT-qPCR quantification. The CV of each reference gene candidate was calculated using the absolute Ct values. CV was used to preliminarily visualize the fluctuation in expression of nine reference gene candidates across sample groups. As shown in Fig. 5, Gluc has the lowest CV at 2.28, closely followed by EIF5A2 at 2.48, EF-hand at 2.61 and GDT1 at 2.69. Interestingly, the two conventional reference genes had either the highest CV (actin at 6.06) or an intermediate CV (NAD5 at 3.41) (Fig. 5).

Three independent statistical analytic tools, GeNorm [33], NormFinder [30], and BestKeeper [88] were used to assess and rank the expressional stability of these nine candidate genes. GeNorm measures the stability of reference gene candidates by calculating gene-stability measure value (*M*). *M* is calculated based on the average pairwise variation of each candidate gene over the other candidate genes. A higher *M* value represents a lower stability of that gene. The least stable candidate gene (having the highest *M* value) is eliminated in a stepwise process. Briefly, after each round of exclusion, *M* value is recalculated for each of the remaining genes and the same pairwise exclusion process is repeated until only two genes remain. NormFinder uses ANOVA-based model to calculate stability value (*SV*) that estimates expressional variations among the tested candidate genes. A higher *SV* indicates a lower stability. NormFinder ranks the stability of candidate genes by taking into account both intra- and intergroup variations. Lastly, BestKeeper assesses stability of candidate genes by considering several parameters, including standard deviation (*SD*), coefficient of variance and Pearson correlation coefficient (*r*). Genes with the highest r value and *SD* < 1 are considered the most stably expressed.

Of the nine candidates tested, three *novel* candidate reference genes (CYSP, NDUFS8 and YLS8) identified in this study were ranked as the most stable by all three algorithms (Table 3). Stability ranking of the rest of the genes differed among tools. For example, the three least stable candidates were EF-hand, actin, and NAD5 based on geNorm; actin, EF-hand, and NAD5 by NormFinder; and NAD5, GDT1, and EF-Hand by BestKeeper (Table 3). It is important to note that both actin and NAD5, two *conventional* reference genes commonly reported in past gene expression literature [4, 91–94], ranked among the least stable by all three algorithms, with the exception that actin was ranked in the middle by BestKeeper.

**Table 3:** Stability rankings of grapevine candidate reference genes across different sample groups (two tissue types, three developmental stages, infected with or free from GLRaV-3) according to geNorm, NormFinder and BestKeeper

Rank	Program						
	geNorm		NormFinder		BestKeeper		
	Gene	*M*	Gene	SV	Gene	SD	r
1	**CYSP**^**1**^	0.277983	**CYSP**^**1**^	0.09	**CYSP**^**1**^	0.6	0.951
2	**NDUFS8**^**1**^	0.277983	**NDUFS8**^**1**^	0.14	**NDUFS8**^**1**^	0.53	0.941
3	**YLS8**^**1**^	0.401338	**YLS8**^**1**^	0.17	**YLS8**^**1**^	0.58	0.879
4	Gluc	0.514505	EIF5A2	0.35	Actin^2^	0.96	0.879
5	EIF5A2	0.535890	Gluc	0.42	Gluc	0.38	0.726
6	GDT1	0.563192	GDT1	0.59	EIF5A2	0.35	0.641
7	EF-hand	0.656230	Actin^2^	0.70	NAD5^2^	0.57	0.562
8	Actin^2^	0.872071	EF-hand	0.76	GDT1	0.35	0.51
9	NAD5^2^	3.809479	NAD5^2^	3.78	EF-hand	0.4	0.335

^1^The three most stable reference gene candidates are bolded. ^2^Two conventionally used reference genes were underscored

*SV* stability value, *SD* standard deviation, *r* Pearson coefficient of correlation

### Comparative analysis of CYSP, actin and NAD5 as reference genes for normalization of UFGT

Anthocyanidin 3-*O*-glucosyltransferase (UFGT) is a key enzyme involved in the biosynthesis of anthocyanins. Two independent studies have reported that UFGT expression was up-regulated in symptomatic leaves but down-regulated in mature berries of dark-skinned grapevine cultivars in response to GLRaV-3 infection [4, 5]. We hypothesized that the use of improper (i.e., less stable) reference genes would lead to incorrect conclusions in RT-qPCR gene expression studies. We performed RT-qPCR analysis on the expression of UFGT in both leaf and berry samples in the context of GLRaV-3 infection (GLRaV-3-infected vs. GLRasV-3 free). For the purpose of comparison, the best-ranking reference gene (CYSP) identified in this study and two of the *conventional* reference genes previously used by other researchers, actin and NAD5, were used to individually normalize UFGT expressional data. As stated earlier, actin and NAD5 were ranked among the least stable genes based on analyses using three different algorithms.

**Fig. 6:**
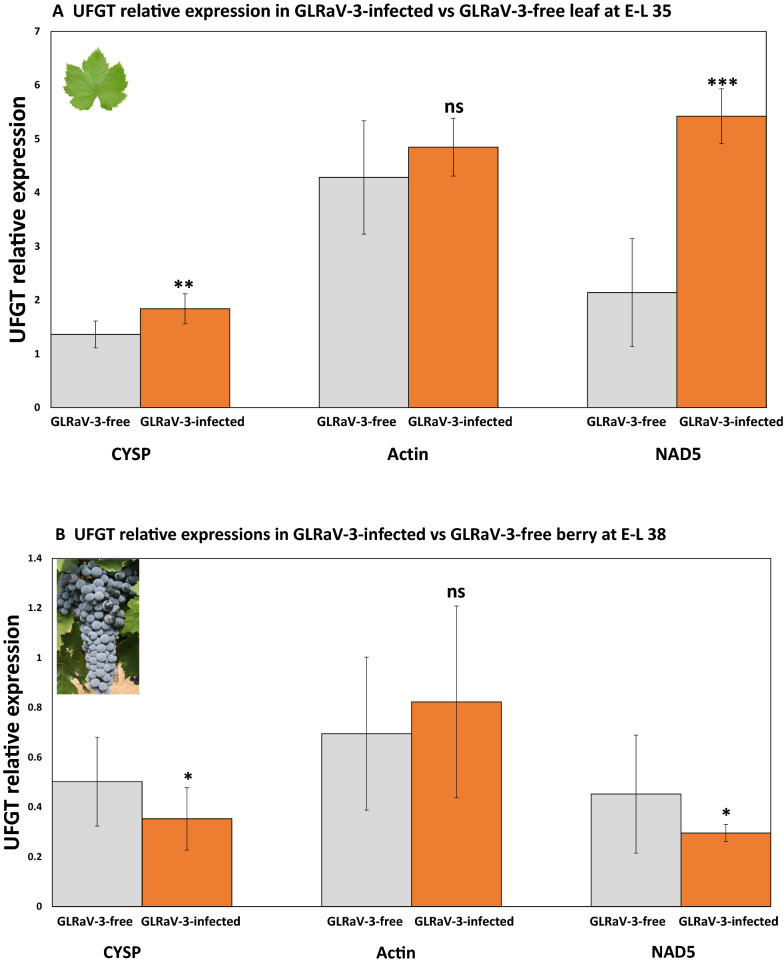
UFGT relative expressions as normalized against three reference genes (CYSP, actin, and NAD5), in GLRaV-3-infected (orange bars) as compared to GLRaV-3-free (grey bars) E-L 35 leaf (**A**) and E-L 38 berry (**B**) of Cabernet Franc. Statistical significance of UFGT expressional change in GLRaV-3-infected vs. GLRaV-3-free samples was evaluated using one-way ANOVA. **A** In Cabernet Franc leaf at E-L 35, dataset normalized to CYSP (left, **P = 0.00768) and to NAD5 (right, ***P = 0.00079) showed that UFGT expression in GLRaV-3-infected leaf was *significantly* different from that in GLRaV-3-free leaf. Data set normalized to actin (middle) showed *no significant difference* between GLRaV-3-infected leaf and GLRaV-3-free leaf (ns, P = 0.23146). **B** In berry at E-L38, data sets normalized to CYSP (left, *P = 0.02235) and to NAD5 (right, *P = 0.03925) showed that UFGT expression was *significantly* different in GLRaV-3-infected samples compared to GLRaV-3-free samples. However, data set normalized to actin (middle) revealed *no significant difference* in UFGT expression due to GLRaV-3-infection (ns, P = 0.77989). UFGT relative expressions were generated by normalizing raw quantification data against each of the three reference genes (CYSP, actin, NAD5). Relative expression values are mean, ± SD (error bars), n = 3. ns: not significant (P > 0.05); *significant (0.01 < P < 0.05); **significant (0.001 < P < 0.01); ***significant (P < 0.001)

First, we wanted to see if the use of less stable reference genes would lead to bias in calling of the *significance* of UFGT expressional change in response to GLRaV-3 infection. Raw UFGT RT-qPCR expression data was normalized individually against CYSP, actin, and NAD5, generating three *relative* expression datasets: CYSP-dataset, actin-dataset, and NAD5-dataset. For each dataset, UFGT relative expressions of GLRaV-3-infected samples were compared to GLRaV-3-free samples using one-way ANOVA. Leaf samples at E-L 35 and berry samples at E-L 38 were chosen for this analysis. In leaf samples, UFGT expression was shown to be *significantly* up-regulated in response to GLRaV-3 infection in both the CYSP-dataset (P < 0.01) and the NAD5-dataset (P < 0.001) (Fig. 6A). Expression of UFGT in berry samples was *significantly* down-regulated in response to GLRaV-3 infection (CYSP-dataset: P < 0.05 and NAD5-dataset: P < 0.05) (Fig. 6B). However, contrary to what was reported in the literature [4, 5], no significant difference was found in UFGT expression between GLRaV-3-infected and GLRaV-3-free samples when the data was normalized to actin (P > 0.05) (Fig. 6A, B).

**Fig. 7:**
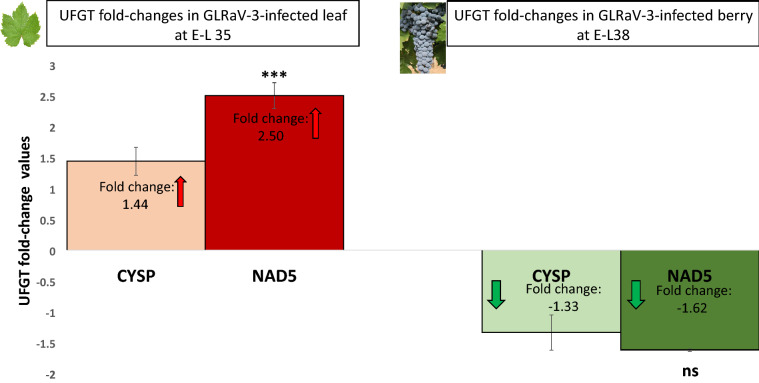
Difference in UFGT fold-change (FC) called in datasets normalized to CYSP and to NAD5 in GLRaV-3-infected leaf at E-L 35 (left), and GLRaV-3-infected berry at E-L 38 (right). Statistical difference between the FC values called in CYSP-dataset and in NAD5-dataset was analyzed using one-way ANOVA. In GLRaV-3-infected leaf (left), UFGT was up-regulated by 1.44-folds when dataset was normalized against CYSP and by 2.50-folds when normalized against NAD5. The FC value from NAD5-dataset was *significantly* different from that from CYSP-dataset (***P = 0). In GLRaV-3-infected berry at E-L 38 (right), UFGT was down-regulated by − 1.33-folds when dataset was normalized against CYSP and by − 1.62-folds when dataset was normalized against NAD5. *No significant difference* was found between the two FC values (ns, P = 0.23303). UFGT fold change values were calculated using Pfaffl method [95]. FC values are mean, ± SD (error bars), n = 3. Positive FC values represent up-regulation of gene (in response to GLRaV-3 infection) and are depicted by red arrows. Negative FC values represent down-regulation (in response to GLRaV-3 infection) and are depicted by green arrows. ns: not significant (P > 0.05); *significant (0.01 < P < 0.05); **significant (0.001 < P < 0.01); ***significant (P < 0.001)

Since UFGT was determined as *significantly differentially* regulated in both datasets normalized to CYSP and to NAD5 (Fig. 6A, B), we further tested if normalization using NAD5 would lead to bias in the calculation of *fold-change (FC)* values. Here, FC values were calculated using the Pfaffl method [95]. We found that in GLRaV-3-infected leaf at E-L 35, UFGT was up-regulated by 1.44-folds when CYSP was used to normalize the data. In contrast, it was substantially up-regulated (at 2.50-folds) when NAD5 dataset was used (Fig. 7). One-way ANOVA analysis revealed that the FC value based on the NAD5-dataset was *significantly* different from that based on the CYSP-dataset in leaf samples (P < 0.001) (Fig. 7, left panel). Therefore, bias was found in calculating the degree of gene expressional change (*FC values*) when a less stable reference gene (i.e., NAD5) was used as compared to the most stable reference gene (i.e., CYSP). In berry samples infected with GLRaV-3, UFGT was *down-regulated* by − 1.33-folds when using CYSP as normalization factor, and by − 1.62-folds when using NAD5 as normalization factor (Fig. 7, right panel). The two FC values were not statistically different from each other (P > 0.05). Based on these results, we conclude that the selection of stable reference genes is essential for the quantification of gene expression studies. As compared to the more stable *novel* reference gene (CYSP), less stable reference genes such as actin and NAD5 would necessarily lead to bias in the calling of both the *significance* and the *degree of fold-change* of grapevine gene expression analysis through RT-qPCR.

## Discussion

### The refined protocol is highly effective in isolating quality RNA from berries of dark-fruited wine grapes

Our lab had previously tackled challenges in RNA isolation from old grapevine leaves with GLRD symptoms [81]. We further refined the protocol by altering parameters involving berry mass and 2-ME concentration at the lysis step in order to overcome challenges in RNA isolation of ripening and mature dark-skinned wine grapes that arose due to increased polyphenolics, polysaccharides, waster, RNases contents and decreased RNA concentration. The optimized RNA isolation protocol we report here differs from previous procedures for RNA isolation from berries by eliminating the need for organic chemicals (phenol and chloroform), significantly shortened assay time (around two hours per batch of RNA isolation) and larger sample size (around twenty-four sample per batch of RNA isolation). We demonstrated that this protocol was suitable for total RNA extraction from leaf and whole berries from a wide range of wine grapes at different phenological stages, including those from vines with viral symptoms. Lastly, we further demonstrated that the resulting RNA preps were suitable for several downstream applications such as RT-PCR, RT-qPCR and RNA-Seq.

### Bioinformatics analyses of RNA-Seq dataset enabled identification of a set of stably expressed genes involved in several biological pathways

Using an established bioinformatics pipeline [86], we have identified 2000 genes from Cabernet Franc as the *core-set candidate reference genes*. Functional enrichment analysis revealed that these candidate genes were largely over-represented in biological activities associated with translation and transcription, including ribosomal assembly, regulation of translation initiation, nucleosome assembly, and nucleotide binding. In addition, some of them were involved in TCA cycle, oxidative phosphorylation, and proteasome-associated protein catabolism (Table 2). Our results were in line with recent studies on wheat [57], rice [96], tomato infected with *Xanthomonas campestris* [45], poplar infected with *Botryosphaeria dothidea* [47], and other organisms such as humans with non-melanoma skin cancers [97], solitary sea squirt [98]*,* blood trematodes [62], and scallop [86]. These studies have all identified their *novel* reference gene candidates to be over-represented in protein synthesis and protein turnover (ubiquitination/proteolysis). Activities associated with transcription, translation, and protein turnover are among the most fundamental cellular processes in all types of organisms. *Novel* reference gene candidates constantly found as over-represented in these cellular activities suggest that these functions may serve as better pools for reference gene selection than the traditionally touted ‘housekeeping’ functions, such as cell structure (actin and tubulin), glycolysis (GAPDH), among others. In fact, it has become increasingly clear that these ‘housekeeping’ genes are heavily regulated under various conditions [35, 99–101].

Some of the *core-set reference gene candidates* identified in our study were also over-represented in respiration process including oxidative phosphorylation and TCA cycle (Table 2 and Additional file 4: Table S6). TCA cycle and oxidative phosphorylation occur in mitochondria and are fundamentally important processes involved in carbohydrate metabolism and ATP production. GLRaV-3 was reported to form viral replication complexes in association with mitochondrial membrane of infected host cells [102–104]. It is hypothesized that GLRaV-3 infections cause damages to mitochondria; and that genes associated with mitochondrial processes would be differentially regulated in GLRaV-3-infected grapevines [70]. Interestingly, results from our study and those reported by others [44, 57, 86, 97] have identified some of the genes involved in respiration as being stably expressed. It is important to note that regulation of gene expression involves a complex network. A subset of genes stably expressed in a biological process cannot be used as evidence to suggest that the said biological process was *non-differentially* regulated under an experimental condition. As a response to GLRaV-3 infection, it is possible that other genes involved in respirational process, not identified among our *2000 core reference genes candidates*, were differentially regulated, consequently, leading to the differential regulation of the general mitochondrial processes. This supposition could be tested by performing proteomic and metabolomic analyses on grapevine infected with GLRaV-3 to examine the regulation of mitochondrial processes at the post-transcriptional level.

### Novel reference genes outperformed traditional reference genes in transcripts quantification

NAD5 and actin were two of the *conventional* reference genes used in gene expression studies involving RT-qPCR [4, 91–94]. They were regarded as the most suitable reference genes in the context of GLRaV-3 infection in grapevine leaf [4]. By using three analytical tools (geNorm, NormFinder, and BestKeeper) commonly used for ranking of gene stability, we compared the stability of seven *novel* reference gene candidates that we have identified in this study with that of NAD5 and actin across two tissue types (leaf and berry), three developmental stages (E-L 31, E-L 35 and E-L 38), and status of GLRaV-3 infection. Results from geNorm, NormFinder and BestKeeper have generated identical rankings on the three most stable reference genes - CYSP, NDUFS8, and YSL8. In stark contrast, actin and NAD5 were ranked among the least stable, suggesting their high levels of variation in expression and hence unsuitability as normalization factors for the quantification of gene expression in grapevine.

In RT-qPCR assays, many of the *conventionally* used reference genes were consistently found to exhibit high expressional variation in various plant species and other organisms [38, 45–47, 57–60, 62, 63]. For example, a recent study by Upadhyay et al. [55] found that actin was the least stable gene in grapevine upon treatment with GA3 application. Gamm et al. [64] and González-Agüero et al. [66], using data from microarray and RNA-Seq respectively, have identified *novel* reference gene candidates in a dark-skinned grapevine cultivar infected with *P. viticola* and *B. cinerea* [64] and genes stably expressed in table grapes at different phenological stages and under abiotic stress [66]. They concluded that all three traditionally used reference genes (18S rRNA, EF1-alpha and UBA10) had higher variation in gene expression when compared to the *novel* reference gene candidates they identified [64, 66]. Our findings were consistent with those from these earlier studies—actin and NAD5 were amongst the most unstable when compared to the seven *novel* reference gene candidates identified in the present study.

The most characteristic symptoms of GLRaV-3-infection in dark-skinned grapevine cultivars are the downward curling of leaf margins and red to purple pigmentation of interveinal regions in leaves [70]. The discoloration of GLRaV-3-infected leaves is associated with increased biosynthesis of anthocyanins, likely resulting from the up-regulation of UFGT gene [4]. In this study, we have revealed that the expression of UFGT was *significantly up-regulated* in leaf tissue at E-L 35 but *significantly down-regulated* in berry at E-L 38 of GLRaV-3-infected Cabernet Franc, when either CYSP or NAD5 was used as a reference gene (Fig. 5A, B). This result was in agreement with two independent studies reported earlier [4, 5]. However, when actin was used as the reference gene, no significant difference was found in UFGT expression between GLRaV-3-infected and GLRaV-3-free samples in either leaf or berry (Fig. 5A, B). Actin has been routinely used by many research groups as a reference gene in gene expression studies [40, 41, 105–113]. Our results raised questions on the validity of conclusions of earlier reports where actin was used as a reference gene in analysis involving grapevine and viral infections. The use of actin for such purposes would lead to false identification of *statistical significance* of a differential regulation, resulting in misinterpretation of data and wrong conclusions to be reached.

Results of analyses using geNorm, NormFinder and BestKeeper were consistent with one another on identifying NAD5, one of the *conventionally* used reference genes, as less stable than CYSP, the *novel* reference gene candidate identified in this study with top stability ranking. When we compared the difference in RT-qPCR results between datasets normalized against NAD5 and CYSP, we found that NAD5 led to bias in calling *FC values*. FC values reveal both the *direction* and *degree* of gene expressional change under an experimental condition. The dataset normalized to NAD5 called UFGT to be up-regulated by 2.50-folds, whereas CYSP-adjusted dataset called UFGT up-regulation by 1.44-folds. These two FC values were drastically different as judged by statistical analysis (Fig. 7). Bias in FC calling not only impact the interpretation of data in terms of the degree of regulation and biological relevance of a differentially expressed gene, but may also skew the decision of researchers as to which gene to select as a priority for further studies. Therefore, skewed results in *significance test* as well as *FC calling* based on the use of improper reference genes, such as actin and NAD5, further emphasize the importance of reference gene selection for use in RT-qPCR quantification studies.

Out results were in agreement with previous studies that *novel* reference genes identified via genome-wide screening outperformed the *conventional* reference genes [38, 45–47, 57–60, 62–64, 66]. This opens discussions for future studies on the potential bias caused by preferentially picking reference gene candidates solely based on those typically used as reported in the literature, without actual evidence to support their suitability as reference genes under the experimental condition in question. In accordance with the recommendation that at least three reference genes should be used for robust normalization of RT-qPCR data [33, 114], we propose the use of CYSP, NDUFS8 and YSL8, for future studies involving the effects of GLRaV-3 infection on grapevine gene expression through RT-qPCR.

## Conclusions

Grape berries are essential to investigations related to fruit chemistry and wine quality. Various molecular assays are employed to assess the expression of genes related to fruit and wine quality and the impact of abiotic and biotic stress factors such as infection with viruses. A key to the success of such assays is an effective protocol that allows sufficient amounts of quality RNA to be obtained from berries collected from veraison and later stages. In this work, we established an optimized protocol through modifications of a total RNA isolation system from Sigma. We have shown that this refined protocol was effective in RNA isolation from older berries of an array of dark-skinned wine grape cultivars, including *V. vinifera* wine grapes and French-American hybrids, resulting in higher yield and integrity. We have further shown that the resulting RNA preps were suitable for downstream applications including amplification of viral RNAs and grapevine genes via RT-PCR, RT-qPCR, and RNA-Seq. We hope that the RNA isolation system we have refined in this work will have utilities in broad research involving grapevine molecular biology, developmental biology, and virus–host interactions.

Using RNA-Seq data derived from leaf and berry of Cabernet Franc at three developmental stages, we have identified three *novel* candidate reference genes for RT-qPCR-based gene expression studies of grapevine: CYSP, NDUFS8, and YSL8. We have shown that these *novel* reference genes were superior over actin and NAD5, two of the *conventional* reference genes previously used in studies involving grapevine and GLRaV-3. Furthermore, we have demonstrated that the choice of improper reference genes for normalization of RT-qPCR data would lead to erroneous conclusions. We anticipate that the three novel reference genes will be useful for studies involving grapevine infections by other viruses involved in the grapevine leafroll disease complex and perhaps many other grapevine viruses. It will be interesting to see if these new reference genes would prove stable under other adverse conditions such as infections by fungal and bacterial pathogens, or insect attack. Naturally, this needs to be tested via experimentation.

## Supplementary Information

**Additional file 1: Table S1.** Total RNA purity and concentration assessment of all samples examined in this study using NanoDrop 1000.

**Additional file 2: Table S2.** Integrity (RIN) and concentration of total RNA from leaf and berry samples of Cabernet Franc as analyzed by Agilent 2100 Bioanalyzer.

**Additional file 3: Table S3.** Candidate reference genes identified in RNA-Seq dataset of leaf samples as ordered by CV values of leaf. **Table S4.** Candidate reference genes identified in RNA-Seq dataset of berry samples as ordered by CV values of berry. **Table S5.** 2000 core candidate reference genes shared by leaf and berry RNA-Seq datasets, ranked by mean CV of CV_leaf and CV_berry.

**Additional file 4: Table S6.** Enriched KEGG pathways of 2000 core candidate reference genes.

## Data Availability

All data generated or analysed during this study are included in this article and its Additional files.
