# Supplementary material for: Genome-wide screening of novel RT-qPCR reference genes for study of GLRaV-3 infection in wine grapes and refinement of an RNA isolation protocol for grape berries
Source: Plant Methods. 2021 Oct 28;17:110. doi: 10.1186/s13007-021-00808-4 (PMC8554853; doi:10.1186/s13007-021-00808-4)
Supplement: Supplementary file 2 — Additional file 2: Table S2. Integrity (RIN) and concentration of total RNA from leaf and berry samples of Cabernet Franc as analyzed by Agilent 2100 Bioanalyzer. [file 13007_2021_808_MOESM2_ESM.docx]

**Table S2**: Integrity (RIN) and concentration of total RNA from leaf and berry samples of Cabernet Franc as analyzed by Agilent 2100 Bioanalyzer.

| **No.** | **Sample Name** | **Nucleic Acid ID** | **Concentration(**ng/μl**)** | **Volume(ul)** | **Total amount(ug)** | **RIN** | **Sample QC Results** |
| --- | --- | --- | --- | --- | --- | --- | --- |
| 1 | EL3154L | CKRN190008575-1A | 286.91 | 32 | 9.18 | 6.5 | Pass |
| 2 | EL31152L | CKRN190008576-1A | 326.27 | 29.8 | 9.72 | 6.2 | Pass |
| 3 | EL31153L | CKRN190008577-1A | 272.78 | 28.5 | 7.77 | 6.3 | Pass |
| 4 | EL3115L | CKRN190008578-1A | 303.83 | 29.3 | 8.9 | 6.4 | Pass |
| 5 | EL3181L | CKRN190008579-1A | 430.19 | 30.3 | 13.03 | 6.4 | Pass |
| 6 | EL3192L | CKRN190008580-1A | 460.86 | 30 | 13.83 | 6.5 | Pass |
| 7 | EL3554L | CKRN190008581-1A | 90.87 | 31.7 | 2.88 | 6.8 | Pass |
| 8 | EL35152L | CKRN190008582-1A | 517.93 | 31.9 | 16.52 | 6.4 | Pass |
| 9 | EL35153L | CKRN190008583-1A | 135.46 | 32 | 4.33 | 6.7 | Pass |
| 10 | EL3515L | CKRN190008584-1A | 354.38 | 31.3 | 11.09 | 6.7 | Pass |
| 11 | EL3581L | CKRN190008585-1A | 194.54 | 29 | 5.64 | 6.8 | Pass |
| 12 | EL3592L | CKRN190008586-1A | 320.95 | 32 | 10.27 | 6.7 | Pass |
| 13 | EL3154B | CKRN190008587-1A | 208.24 | 29.7 | 6.18 | 9.8 | Pass |
| 14 | EL31152B | CKRN190008588-1A | 141.96 | 31 | 4.4 | 9.8 | Pass |
| 15 | EL31153B | CKRN190008589-1A | 305.48 | 30.8 | 9.41 | 9.9 | Pass |
| 16 | EL3115B | CKRN190008590-1A | 279.43 | 30.1 | 8.41 | 9.9 | Pass |
| 17 | EL3181B | CKRN190008591-1A | 289.76 | 31.1 | 9.01 | 9.7 | Pass |
| 18 | EL3192B | CKRN190008592-1A | 144.26 | 29.5 | 4.26 | 9.8 | Pass |
| 19 | EL3554B | CKRN190008593-1A | 76.15 | 30.4 | 2.32 | 7 | Pass |
| 20 | EL35152B | CKRN190008594-1A | 188.82 | 27.7 | 5.23 | 7 | Pass |
| 21 | EL35153B | CKRN190008595-1A | 162.78 | 29.8 | 4.85 | 8.2 | Pass |
| 22 | EL3515B | CKRN190008596-1A | 92.51 | 29.8 | 2.76 | 8.2 | Pass |
| 23 | EL3581B | CKRN190008597-1A | 199.94 | 31.7 | 6.34 | 7.7 | Pass |
| 24 | EL3592B | CKRN190008598-1A | 144.67 | 32.2 | 4.66 | 8.4 | Pass |
| 25 | EL3854B | CKRN190008599-1A | 131.86 | 32.5 | 4.29 | 9.1 | Pass |
| 26 | EL38152B | CKRN190008600-1A | 166.18 | 31.4 | 5.22 | 9 | Pass |
| 27 | EL38153B | CKRN190008601-1A | 169.63 | 32.2 | 5.46 | 8.9 | Pass |
| 28 | EL3815B | CKRN190008602-1A | 169.44 | 32.3 | 5.47 | 8.8 | Pass |
| 29 | EL3881B | CKRN190008603-1A | 125.16 | 31.5 | 3.94 | 9 | Pass |
| 30 | EL3892B | CKRN190008604-1A | 234.89 | 31.1 | 7.31 | 7.7 | Pass |
